# Phenotypic characterization of regional human meniscus progenitor cells

**DOI:** 10.3389/fbioe.2022.1003966

**Published:** 2022-10-19

**Authors:** Jingsong Wang, Sally Roberts, Weiping Li, Karina Wright

**Affiliations:** ^1^ Department of Orthopedics, Sun Yat-sen Memorial Hospital, Sun Yat-sen University, Guangzhou, China; ^2^ Spinal Studies & Cartilage Research Group, Robert Jones and Agnes Hunt Orthopaedic Hospital NHS Trust, Oswestry, United Kingdom; ^3^ School of Pharmacy and Bioengineering, Keele University, Staffordshire, United Kingdom

**Keywords:** progenitor cells, meniscus, cartilage, fibronectin, avascular, vascular

## Abstract

Stimulating meniscus regeneration using meniscal progenitor cells has been suggested as a promising new strategy. However, there is a lack of studies which decisively identify and characterize progenitor cell populations in human meniscus tissues. In this study, donor-matched progenitor cells were isolated *via* selective fibronectin adhesion from the avascular and vascular regions of the meniscus and chondroprogenitors from articular cartilage (n = 5). The mixed populations of cells from these regions were obtained by standard isolation techniques for comparison. The colony formation efficacy of avascular progenitors, vascular progenitors and chondroprogenitors was monitored using Cell-IQ^®^ live cell imaging. Proliferation rates of progenitors were compared with their mixed population counterparts. Cell surface markers indicative of mesenchymal stromal cells profile and progenitor markers were characterized by flow cytometry in all populations. The fibrochondrogenic capacity was assessed *via* fibrochondrogenic differentiation and measuring GAG/DNA content and morphology. All meniscal progenitor and chondroprogenitor populations showed superior colony forming efficacy and faster proliferation rates compare to their mixed populations. Progenitor populations showed significantly higher positivity for CD49b and CD49c compared to their mixed population counterparts and chondroprogenitors had a higher positivity level of CD166 compared to mixed chondrocytes. GAG/DNA analysis demonstrated that progenitor cells generally produced more GAG than mixed populations. Our study demonstrates that the human meniscus contains meniscal progenitor populations in both the avascular and vascular regions. Meniscal progenitors derived from the vascular region exhibit enhanced proliferative and fibrochondrogenic characteristics compared to those from the avascular region; this may associate with the enhanced meniscal healing potential in the vascular region. These findings build on the body of evidence which suggests that meniscal progenitors represent an attractive cell therapy strategy for meniscal regeneration.

## Introduction

Menisci play a key role in joint congruence, dispersing load and protecting the articular cartilage surface of the femur and tibia (Fox et al., 2012). Although treatment of meniscal tears has developed dramatically *via* treatments such as meniscus repair and replacement strategies, these surgical interventions provide limited protection against the progression of osteoarthritis (OA) (Rath and Richmond, 2000; Hommen et al., 2007). Therefore, there is a demand to develop other novel treatments to improve meniscus repair and prevent or delay the onset and progression of OA.

The meniscus is composed of an outer “vascularized” zone that contains elongated fibroblast-like cells and an inner “vascularized” zone that contains rounded fibrochondrocytes. It has long been known that in the vascular region, tears of the meniscus tend to successfully repair themselves after a surgical procedure, whereas the inner avascular region has a low healing potential (Shimomura et al., 2018). Mobilization and homing of endogenous progenitor cells from the vascular zone of the meniscus may be responsible for some of the natural healing noted in the tissue following injury (Guo et al., 2018). Indeed the enhanced regeneration of the vascular region of the meniscus might be due to the presence of CD34 and CD166 immunopositive progenitor cells visible *via* histological analysis in the blood vessels (Osawa et al., 2013). Further studies suggest the presence of progenitor cells in the meniscus promote repair of injured menisci in bovine, rabbit and mouse models (Huang et al., 2016; Gamer et al., 2017; Seol et al., 2017). Our previous work suggested that the “tree-like” collagen structure in the vascular region may contained a source of progenitor cells (Wang et al., 2020), but there is a lack of studies that decisively identify and characterize progenitor cell populations in different regions of the human meniscus.

Fibronectin-coated flasks have been commonly used to extract chondroprogenitors from articular cartilage and these progenitors have been investigated for their capability in terms of cartilage regeneration (Williams et al., 2010). Chondroprogenitors are an ideal candidate for cell-based tissue engineering cartilage repair strategies because they are known as a relatively undifferentiated population of chondrocyte precursors, which are less likely to become hypertrophic and terminally differentiate sooner than their counterpart mature chondrocytes (Jayasuriya and Chen, 2015). In this study, an established chondroprogenitor isolation protocol (Williams et al., 2010) was used to obtain and characterize the progenitors and whole mixed populations from donor-matched avascular and vascular regions of the meniscus, as well as the two cellular fractions from articular cartilage derived from the same individual.

## Materials and methods

### Patient demographics

**FIGURE 1:**
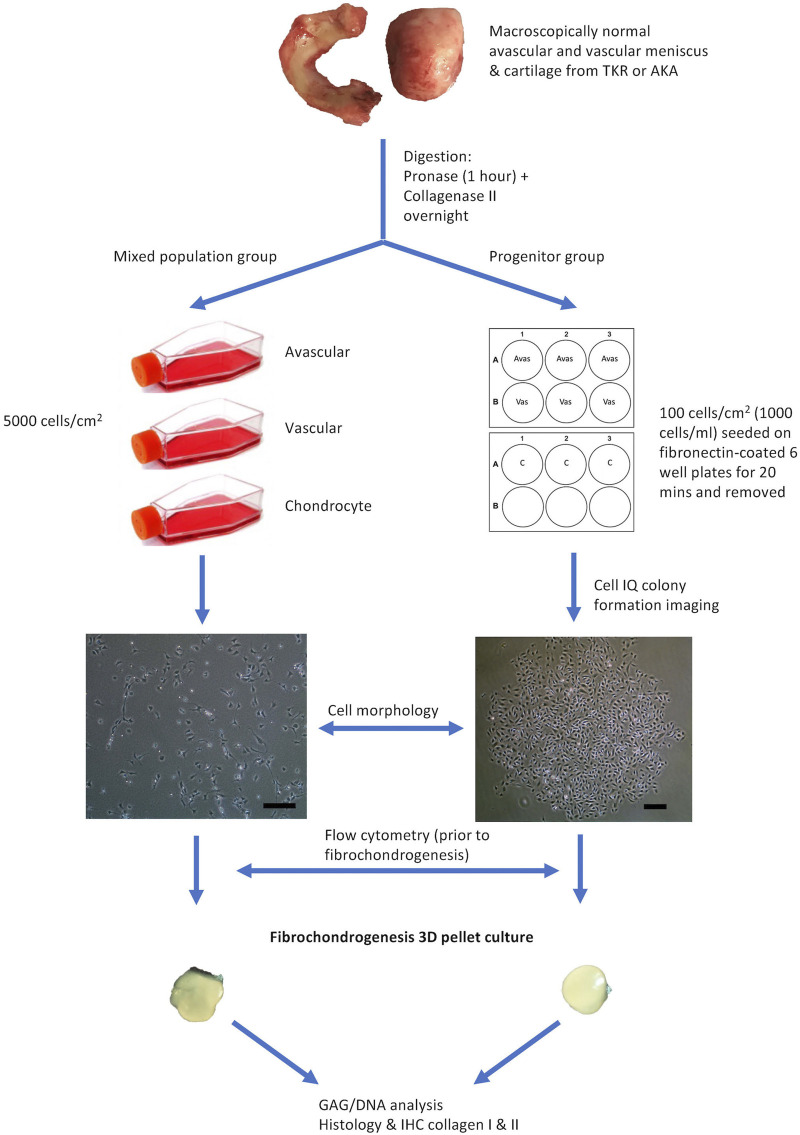
Flow Diagram Schematic of the Experimental Plan. Avas: Avascular meniscal cells, Vas: Vascular meniscal cells, C: Chondrocytes, TKR: total knee replacement, AKA: above-knee amputation, IHC: immunohistochemistry. Scale bars represent 250 μm.

The provision of written informed consent was obtained from each patient prior to surgery. Favorable ethical approval was given by the National Research Ethics Service (NRES number 11/NW/0875) and all experiments were performed following relevant guidelines and regulations. Macroscopically, intact lateral menisci from the lateral compartment (five patients), as well as donor-matched articular cartilage from the lateral femoral condyle (four patients, except patient 2), which demonstrated minimal OA changes (macroscopically classified as Outerbridge Grade I or II), were harvested from four donors who were undergoing TKR for medial compartment and one patient undergoing above-knee amputation (Table 1). The general workflow is shown in Figure 1.

**TABLE 1:** Patient demographics.

Patient	Age	Sex	Procedure	Tissue type	Additional clinical information
1	37	male	TKR	Lateral meniscus; cartilage from LFC	Previous HTO, medial artificial meniscus and meniscus allograft transplant. Has received multiple knee wash outs and removal of osteophytes
2	73	male	TKR	Lateral meniscus*	Advanced OA with bone-on-bone in the medial compartment
3	60	female	TKR	Lateral meniscus; cartilage from LFC	Indication of OA from imaging assessments
4	65	male	TKR	Lateral meniscus; cartilage from LFC	Bone-on-bone medial compartment OA, presence of significant osteophytes
5	59	female	AKA	Lateral meniscus; cartilage from LFC	Previous traffic accident, osteoporosis due to immobility

TKR: total knee replacement; AKA: above knee amputation; OA: osteoarthritis; HTO: high tibial osteotomy; LFC: lateral femoral condyle. * Only avascular and vascular meniscus tissue were taken from patient two.

### Progenitor cell isolation

The meniscus was dissected longitudinally into three parts: the inner avascular zone, the middle and the outer vascular zone. The middle portion was discarded and only the extreme inner and outer avascular and vascular zones were used to derive meniscal cells in order to ensure distinct regions. Additionally, full-depth macroscopically normal human articular cartilage from femoral condyles was used to isolate chondrocytes and their progenitors. Samples were digested by sequential pronase (70 U/ml, 1 h at 37°C) and collagenase type II (245 U/ml, 12 h at 37°C; Worthington, United States) incubation in Dulbecco’s Modified Eagle Medium (DMEM)/F12 (1:1) (Gibco, United States), 1% penicillin–streptomycin (P/S) (ThermoFisher Scientific, United States) and 1% Insulin-Transferrin-Selenium-X (ThermoFisher Scientific, United States). Cells extracted from the three tissue types (avascular and vascular menisci and articular cartilage) were cultured under two conditions. The first was for the isolation and growth of a mixed population of cells (avascular meniscal mixed cells (MAvas), vascular meniscal mixed cells (MVas) and mixed chondrocytes (MChs)), which were each plated at a density of 5,000 cells/cm^2^. The second was for the isolation and growth of progenitor cells (avascular meniscal progenitors (PAvas), vascular meniscal progenitors (PVas) and chondroprogenitors (PChs)), which were subjected to a fibronectin selective adhesion as previously described (Williams et al., 2010). Briefly, six well plates were coated with 10 μg/ml fibronectin (Sigma-Aldrich, United Kingdom) in phosphate buffered saline (PBS), containing 1 mM MgCl_2_ and 1 mM CaCl_2_ overnight at 4°C. Isolated mixed populations from each tissue fraction were seeded onto the coated wells at a low density of 100 cells/cm^2^ (1,000 cells/ml) in triplicate for 20 min at 37°C in DMEM/F12 medium, 10% Foetal Bovine Serum (FBS) (Gibco, United States), 1% P/S and 0.1 mM ascorbic acid (Sigma-Aldrich, United Kingdom) (culture medium). After 20 min, medium and non-adherent cells were removed and fresh culture medium was added to the remaining adherent cells and incubated at 37°C, 5% CO_2_.

### Cell-IQ^®^ live cell imaging

After 3 days of culture in a six well plate, progenitor cells were imaged using a Cell-IQ^®^ phase contrast Live Imaging Platform (CM Technologies, Tampere, Finland) to monitor colony formation. Spare wells and surrounding areas in six well plates were filled with distilled water to keep the plate humidified. At least one colony was monitored in each well and each colony was imaged every 30 min for 48 h. The recorded images were analyzed using the Cell-IQ^®^ Analyzer (CM Technologies, Tampere, Finland) software in order to measure the live cell number in each colony every 30 min.

### Growth kinetics

The number of monoclonal colonies (defined as a cluster of more than 32 cells which represents a population of cells derived from more than five population doublings of a single cell) in each well was counted under light microscopy after 5 days of culture, which was considered as the initial number of progenitors that had adhered to the plate (Williams et al., 2010). Each type of progenitor population in each well was then trypsinised by 0.05% trypsin/EDTA (Gibco-Thermo Fisher Scientific, United States) and reseeded in a T25 tissue culture flask (Sarstedt, Germany) in culture medium supplemented with 1 ng/ml transforming growth factor beta 1 (TGF-β1) (PeproTech, United Kingdom), 5 ng/ml fibroblast growth factor 2 (FGF-2) (PeproTech, United Kingdom). Population doubling times (PDTs) were calculated using the formula: PDT = (t_2_-t_1_) x ln (2)/ln (n_2_/n_1_), where t_1_ = the time of cell seeding, t_2_ = the time of cell harvest and n = the cell population at the matching time points. PDTs at passage 0 to 2 were compared between mixed populations and progenitor cells, as progenitors could not be counted with sufficient accuracy at P0-1.

### Flow cytometry

Prior to fibrochondrogenic differentiation, 1,20,000 avascular meniscal cells, vascular meniscal cells and chondrocytes from both mixed population and progenitor groups were resuspended in a PBS buffer consisting of 2% (w/v) bovine serum albumin (BSA; Sigma-Aldrich). Non-specific antibody binding was blocked using a PBS buffer composed of 10% (v/v) human immunoglobulin (Grifols, Spain) at 4°C for 1 h. Immunopositivity for nine surface molecules which are indicative of mesenchymal stromal cells (MSCs) the International Society for Cellular Therapy (ISCT) (Dominici et al., 2006) profile (CD14, CD73, CD90, CD105), progenitor markers (CD44 CD166) or cell adhesion molecules (CD29, CD49b, CD49c) were evaluated in all five donors.

### Fibrochondrogenic differentiation assay

The fibrochondrogenic potential of the donor-matched populations in both mixed populations and progenitor groups was assessed at passage two using a well-established 3D pellet culture protocol (Yoo et al., 1998) in five donors (with the exception of the mixed and progenitor chondrocyte populations from patient 2). Briefly, 2.5 × 10^5^ cells were centrifuged into a cell pellet with DMEM/F12, 1% P/S, 1% insulin transferrin selenium (Sigma-Aldrich, United Kingdom), 1 mM l-ascorbic acid-2-phosphate (Sigma-Aldrich, United Kingdom), 10 nM dexamethasone, 1 mM sodium pyruvate (Sigma-Aldrich, United Kingdom) and 10 ng/ml TGF-β1 (PeproTech, United Kingdom). After 28 days in culture, three pellets from each donor and cell type were used for biochemical quantitation of GAG/DNA and snap frozen in liquid nitrogen-cooled hexane and stored at −80°C until histological analysis.

### GAG/DNA analysis

After 28°days in culture, three pellets of each cell type were digested in papain to release GAG and DNA. The papain digestion buffer (125 μg/ml, pH 6.0) was composed of 50 mM sodium phosphate (BDH), 20 mM EDTA (Sigma-Aldrich), 20 mM N-acetyl cysteine (BDH), papain (Sigma-Aldrich). Each pellet was digested in 200 µL of the papain digestion buffer at 60°C for 3 h, after which all samples were centrifuged at 1000 g for 5 min and supernatants were stored at −20°C prior to use. The glycosaminoglycans (GAG) in pellets were assessed by 1,9-dimethylmethylene blue (DMMB) assay (FARNDALE et al., 1986). Briefly, 50 μL of each sample and 200 μL of DMMB solution were added in triplicate to a 96 well plate. The results were read immediately at A530 nm and A590 nm using a FluorStar Omega microplate reader (BMG Labtech, Ortenberg, Germany). The bovine chondroitin sulphate (C9819, Sigma-Aldrich) was used to construct a standard curve, which was plotted using the following equation: (A530 nm/A590 nm)- (A530 nm blank/A590 nm bank). The total GAG content of each pellet was calculated from the standard curve. The DNA content was measured spectrofluorometrically using a PicoGreen dsDNA Assay kit (Invitrogen) according to the manufacturer’s instructions. Finally, the GAG content was normalised to the corresponding DNA content per pellet.

### Sectioning and immunohistochemical staining of fibrochondrogenic pellets

Three pellets from each cell population were cryosectioned at 7 μm thickness. Cryosections were immunohistochemically stained for collagen types I and II. In brief, sections were pre-treated with hyaluronidase (4800 U/ml, Sigma, United Kingdom) for 2 h and fixed in 10% formalin for 10 min. Sections were then washed with PBS and incubated with primary mouse collagen type I antibody (1:500, clone I-8H5, MP Biomedicals, Cambridge, United Kingdom) and collagen type II antibody (1:50, clone CIIC1, DHSB, University of Iowa, United States) in PBS for 1 h. Negative control sections were incubated with nonspecific, isotype matched antibodies (for collagen type I: IgG2a; for collagen type II: IgG1, Dako, Denmark) instead of primary antibodies at the same concentration. Sections were then washed in PBS before incubation with the secondary biotinylated antibody at 50 μg/ml (goat anti-mouse, VECTASTAIN ABC kit, Vector Laboratories, Peterborough, United Kingdom) for 30 min. Hydrogen peroxide [0.3% (v/v)] in methanol (BDH) was used to eliminate endogenous peroxidase activity. Labelling with streptavidin-peroxidase was enhanced with incubation of an avidin-biotin complex (VECTASTAIN Elite ABC kit, Vector Laboratories, United Kingdom) for 30 min according to the manufacturer’s instructions. After washing with PBS, sections were visualised with diaminobenzidine (DAB, ImmPACT, Vector Laboratories, Peterborough, United Kingdom) and then dehydrated before mounting under glass coverslips with Pertex mounting medium. The immunohistochemistry staining intensity of collagen type I and type II was semi-quantified by a previously established protocol using ImageJ Fiji Software (version 1.2; WS Rasband, National Institute of Health, Bethesda, MD) (Crowe and Yue, 2019).

### Statistical analyses

GraphPad Prism (Version 8.30, San Diego, California, United States) was used for statistical analysis. Two-way ANOVA with a multiple comparisons test was used to analyse flow cytometry, population doubling time, pellet GAG/DNA assay and pellet collagen staining intensity. Data were presented as mean ± standard deviation (SD) in the graphs and text.

## Results

### Growth kinetics and cell morphology in mixed population and progenitor cells

**FIGURE 2:**
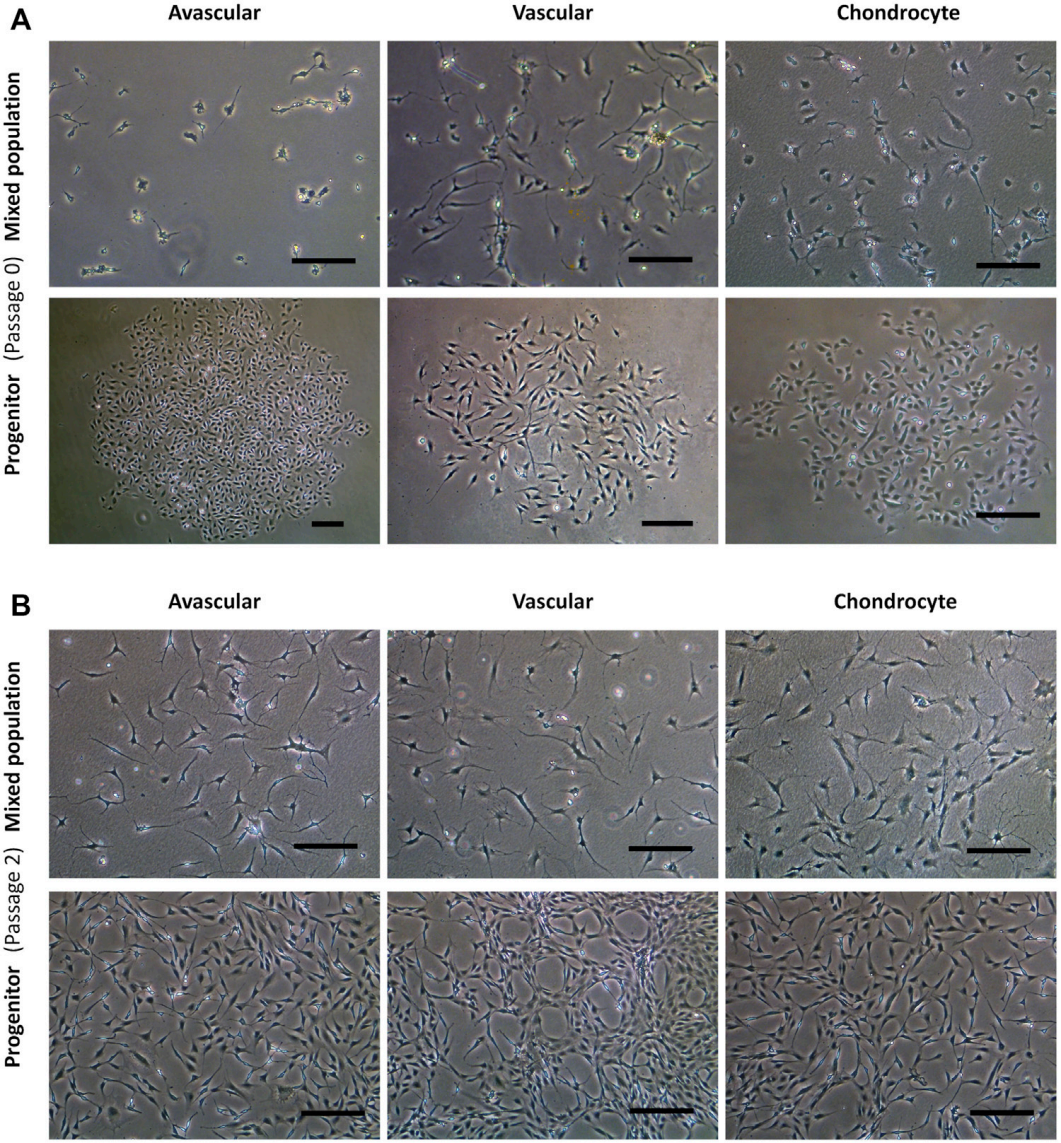
Comparison of progenitor cells morphologies isolated from meniscal and cartilage tissues in monolayer culture at passage 0 and 2. Representative images from a single donor for mixed population and progenitor cells at passage 0 **(A)** and passage 2 **(B)** including avascular and vascular meniscal cells and chondrocytes (left to right). Scale bars represent 250 μm.

Figure 2 shows the representative mixed and progenitor cell population morphologies. At passage 0 (Figure 2A), mixed population cells were distributed randomly on tissue culture plastic, whereas progenitor cells were organised into round tightly packed colonies. PAvas and PChs had a similar oval chondrocyte-like morphology whereas PVas were more fibroblast-like and spindle-shaped in appearance. At passage 2 (Figure 2B), the majority of the mixed population cells possessed extensive cytoplasmic processes of varying length (Bush and Hall, 2003), but these were much less common in progenitor cell populations.

**FIGURE 3:**
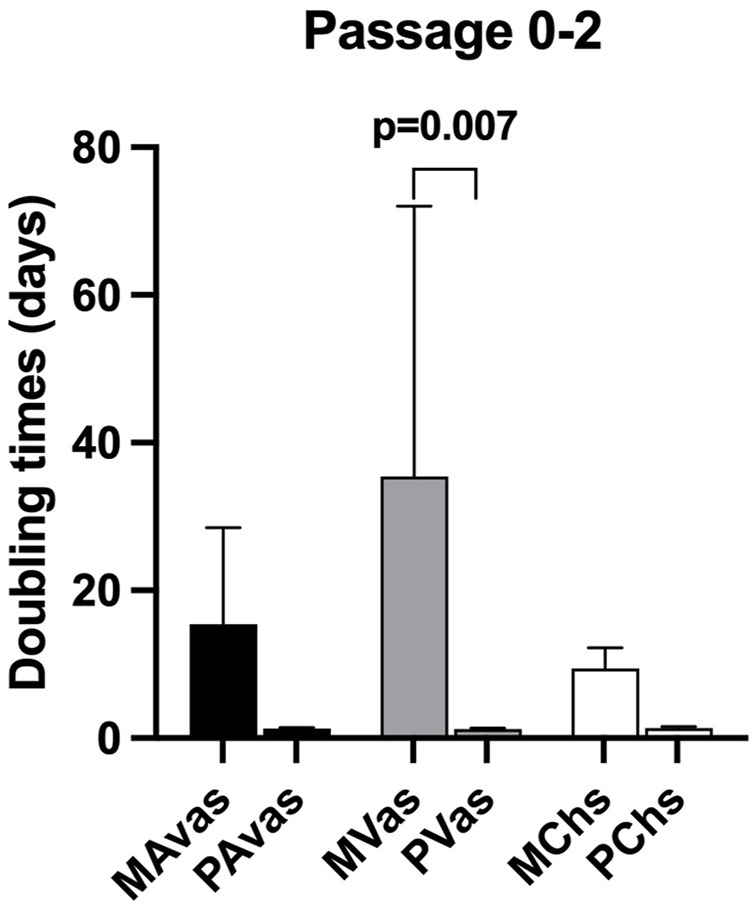
Population doubling time (PDT) data for progenitor and mixed cell populations. MAvas: mixed avascular meniscal cells, MVas: mixed vascular meniscal cells, MChs: mixed chondrocytes. PAvas: progenitor avascular meniscal cells, PVas: progenitor vascular meniscal cells, PChs: progenitor chondrocytes. Data are presented as mean ± standard deviation.

Mixed populations demonstrated a slower growth rate in monolayer culture compared to progenitor cells at passages 0 to 2. The mean PDT of MAvas, MVas and MChs at P0-2 were 15.46 ± 13.05 days, 35.40 ± 36.62 days and 9.44 ± 2.78 days respectively compared with 1.27 ± 0.14 days, 1.25 ± 0.11 days and 1.31 ± 0.18 days for PAvas, PVas and PChs, respectively. However, the only statistically significant difference was found between MVas and PVas at P0-2 (*p* = 0.007) (Figure 3).

### Cell-IQ^®^ live cell imaging analysis

**FIGURE 4:**
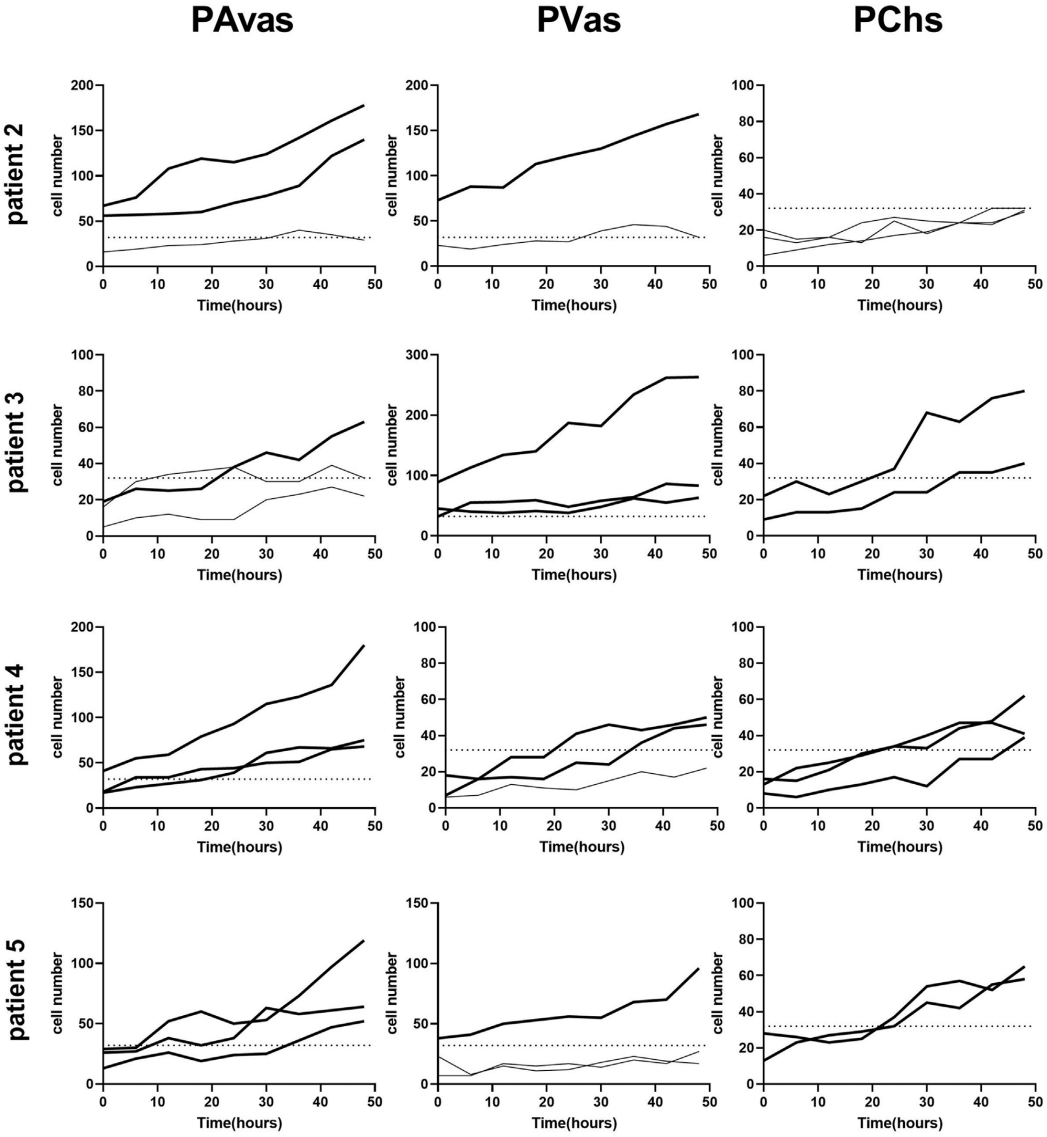
Diagram of individual colony proliferation rates over 48 h in the Cell-IQ^®^. Progenitor colonies (cell numbers beyond 32) are indicated in bold. The dashed lines represent the threshold of the minimum cell number considered to be a progenitor colony (n = 32). PAvas: progenitor avascular meniscal cells, PVas: progenitor vascular meniscal cells, PChs: chondroprogenitors.

**FIGURE 5:**
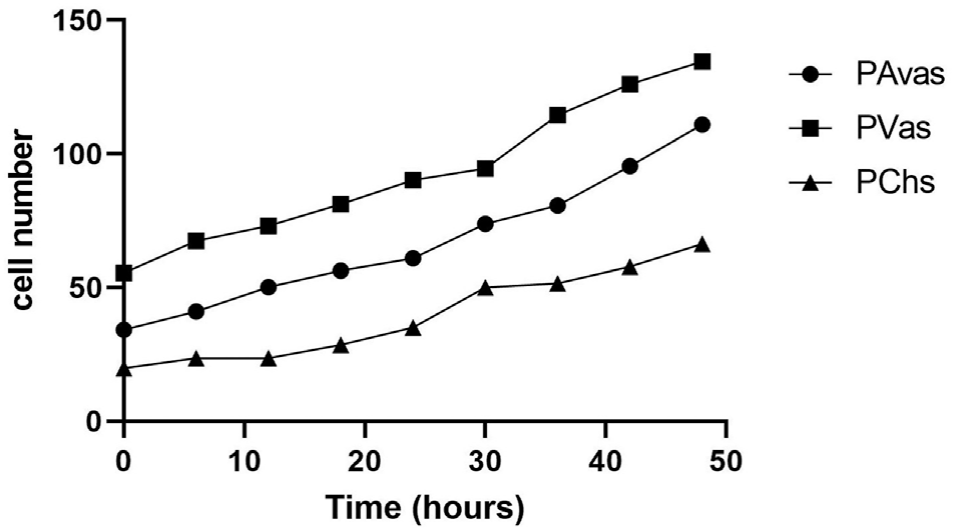
The comparison of colony proliferation rate (colony cell >32 cells) for avascular (PAvas) (n = 7), vascular meniscal progenitors (PVas) (n = 7) and chondroprogenitors (PChs) (n = 6). Date showed the mean cell number of progenitor colonies in every 6 h.

Four of five patients’ progenitor colonies of PAvas, PVas and PChs were monitored for 48 h in the Cell-IQ^®^ live cell imager. For each progenitor cell type from each patient, two or three colonies were selected for colony proliferation rate analysis. Figure 4 showed the results of individual colony proliferation data. Colonies with less than 32 cells beyond 48 h in culture were not considered to be progenitor colonies and so were excluded; these comprised three of 12 colonies for PAvas, four of 11 colonies for PVas and three of 10 colonies for PChs which were characterized as non-progenitor colonies (Figure 4). After excluding these non-progenitor colonies (10 of 33 colonies, 30.3%), proliferation data from progenitor colonies only was compared for PAvas, PVas, PChs fractions (Figure 5). The comparison analyses (Table 2) showed that PVas colonies proliferated significantly faster than PAvas (*p* = 0.0022) and PChs (*p* < 0.0001), whilst the PChs colony proliferation rate was significantly slower than the rate of proliferation for PAvas colonies (*p* = 0.0026). The representative colony proliferation videos (donor 3) of PAvas, PVas and PChs were uploaded in the supplement material.

**TABLE 2:** Multiple comparison tests for proliferation rates of progenitor colonies.

	Predict means, 95% CI	*p* value
PAvas vs. PVas	(67.00, 92.98), (-43.83 to -8.127)	0.0022
PAvas vs. PChs	(67.00, 39.53), (8.298–46.65)	0.0026
PVas vs. PChs	(92.98, 39.53), (32.45–74.45)	<0.0001

PAvas: progenitor avascular meniscal cells, PVas: progenitor vascular meniscal cells, PChs: chondroprogenitors, CI: confidence interval.

### Cell surface marker immunoprofiles

**FIGURE 6:**
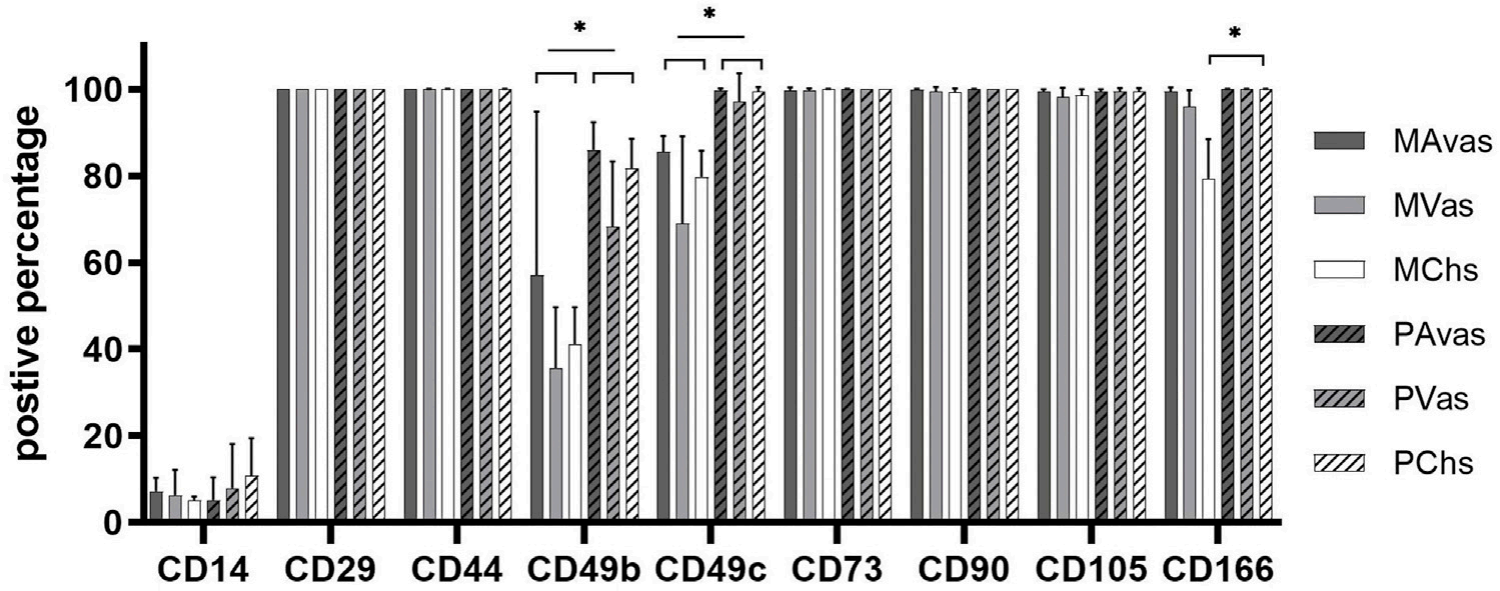
Meniscus and cartilage derived mixed cell and progenitor immunoprofiles; Histograms showing the percentage of cells with immunopositivity for cell surface markers on donor matched mixed or progenitor avascular and vascular meniscal cells and chondrocytes at passage 2–3. Data were presented as mean ± standard deviation (n = 5).

Flow cytometry analyses (Figure 6) revealed that all cell populations were over 95% immunopositive for the ISCT MSC markers CD73, CD90 and CD105, as well as other matrix adhesion markers (CD29 and CD44). CD14 was present on all cell populations, ranging on average from 5.18% to 10.75% positivity. There was no significant difference noted for any of these cell surface markers when comparing mixed populations and progenitor cells. However, differences between cell types for the integrins CD49b, CD49c and the chondrogenic potency marker CD166 (Brinkhof et al., 2020) were noted. Progenitor cells showed significantly higher positivity for CD49b compared to their counterpart mixed avascular meniscal fractions (*p* < 0.0001), vascular meniscal cells (*p* < 0.0001) and chondrocytes (*p* < 0.0001). Interestingly, both MAvas and PAvas had significant greater positivity for CD49b compared to MVas (*p* = 0.0002) and PVas (*p* = 0.0035), respectively. Progenitor cells also showed a significantly increased level of CD49c when compared to their paired mixed populations in avascular meniscal (*p* = 0.0387), vascular meniscal (*p* < 0.0001) and chondrocyte fractions (*p* = 0.0035). MAvas showed significantly higher positivity for CD49c compared to MVas, whereas no difference was noted in progenitor cell types. For CD166, MChs had a significantly lower positivity compared to MAvas (*p* = 0.0012), MVas (*p* = 0.0144) and PChs (*p* = 0.0019).

### *In Vitro* fibrochondrogenic differentiation analysis

**FIGURE 7:**
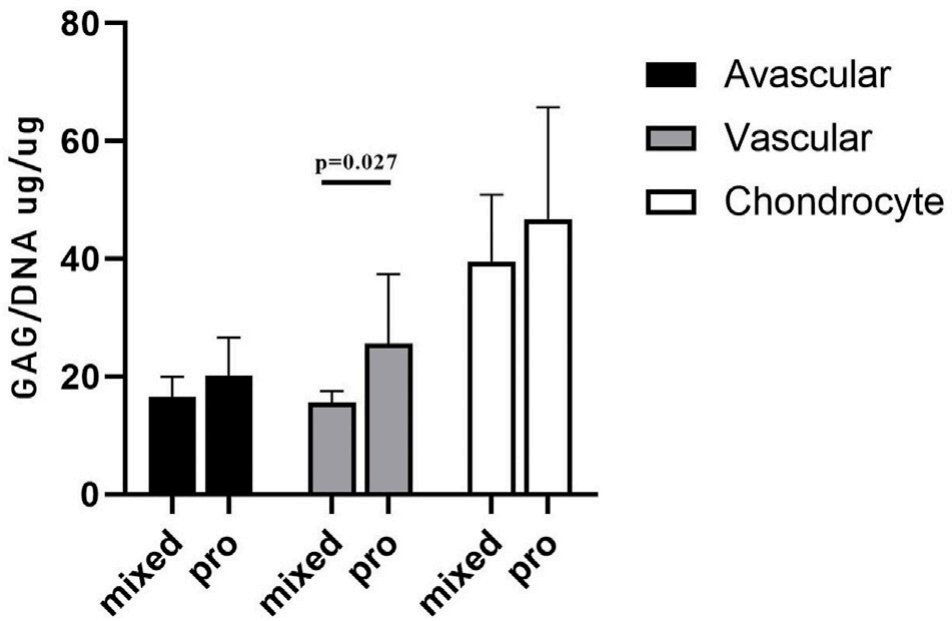
GAG/DNA quantitation after 28 days of 3D pellet culture. Comparison of mixed populations and progenitor (pro) avascular and vascular meniscal cells and chondrocytes from five donor matched samples. Data were presented as mean ± standard deviation (n = 3 pellets/donor).

**FIGURE 8:**
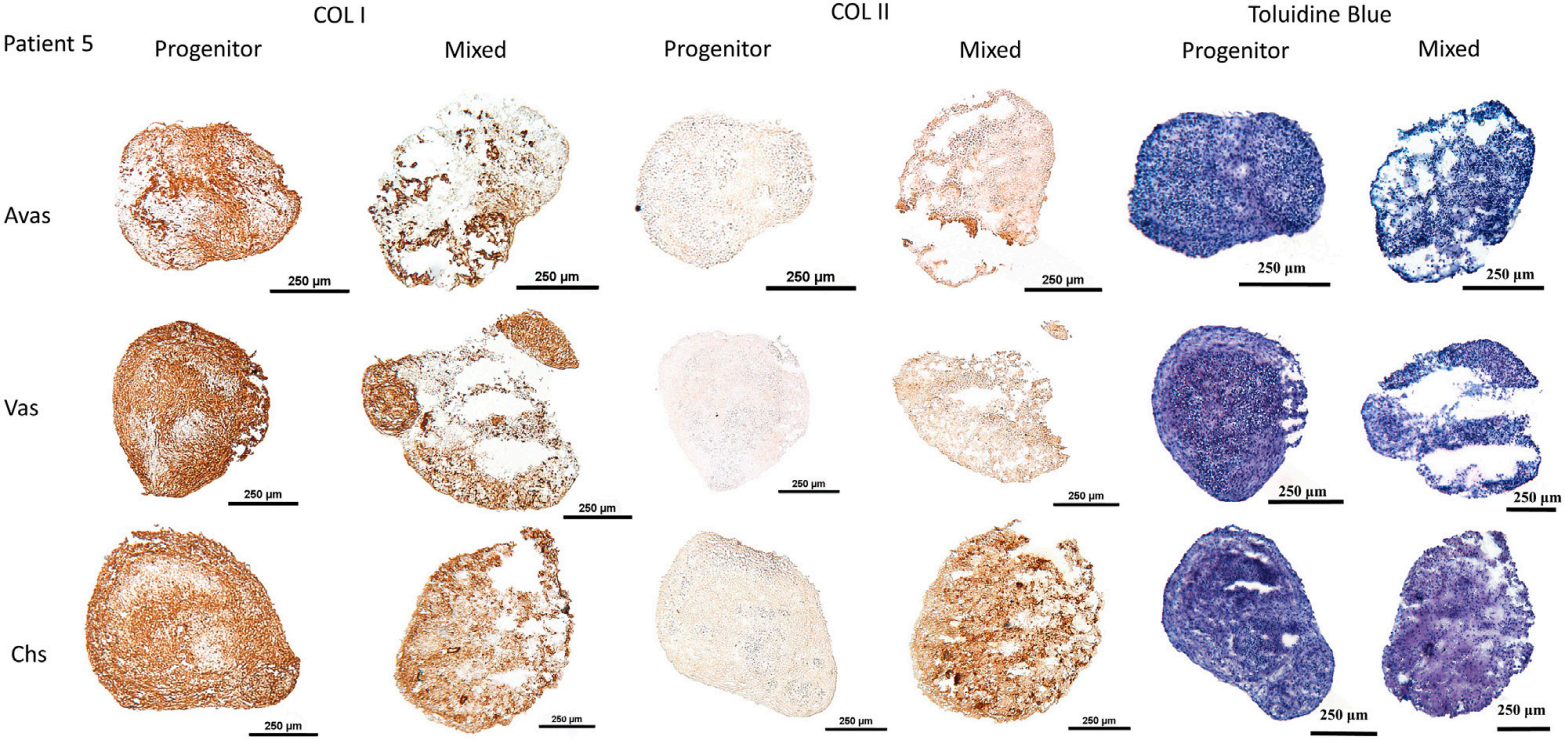
Chondrogenesis of both mixed population and progenitor cells from avascular and vascular meniscus and cartilage from a representative patient 5, assessed by staining sections using type I and type II collagen immunohistochemistry and toluidine blue metachromatic staining of GAGs. Calibration bars = 250 μm. Avas: Avascular meniscal cells; Vas: Vascular meniscal cells; Chs: Chondrocytes.

**FIGURE 9:**
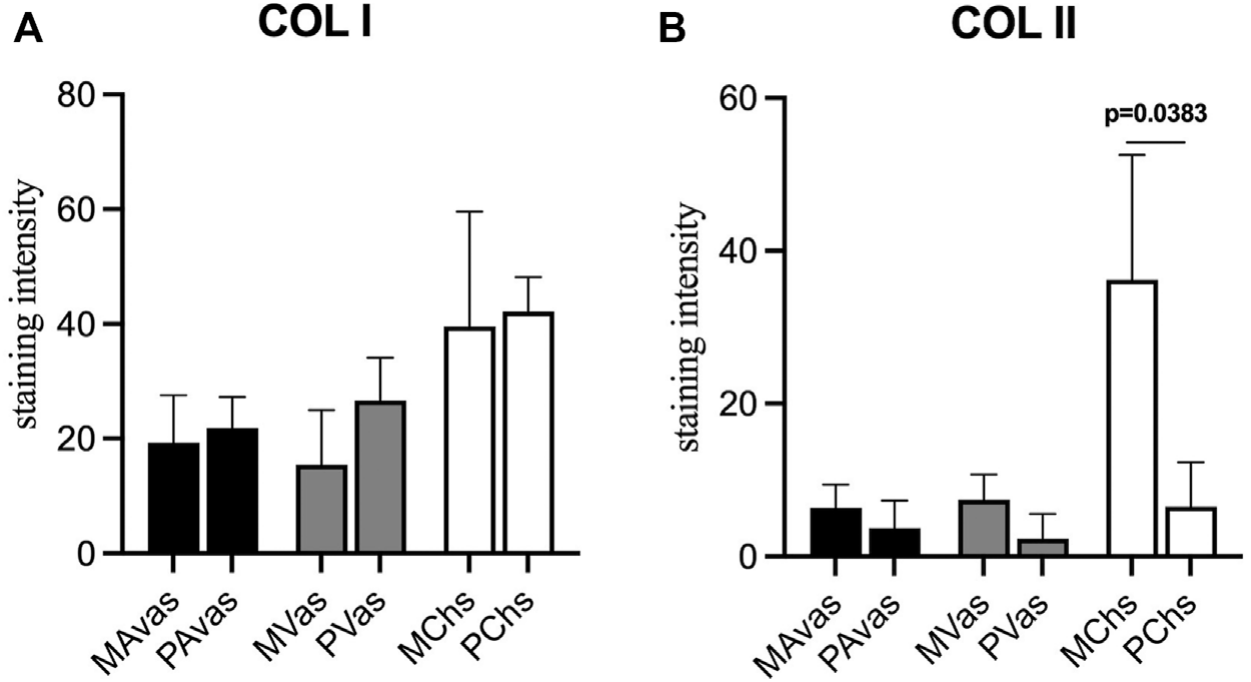
Semi-quantitative immunohistochemical staining measurement for collagen type I **(A)** and collagen type II **(B)** of mixed population (MAvas, MVas, MChs) and progenitor (PAvas, PVas, PChs) avascular and vascular meniscal cells and chondrocytes pellets. Data were presented as mean ± standard deviation.

GAG/DNA analysis (Figure 7) demonstrated that progenitor cells generally produced more GAGs than mixed population cells derived from all three tissues, with significant differences noted between MVas and PVas (*p* = 0.027). The GAG/DNA level of chondrocytes (mixed and progenitor) were also significantly higher than avascular and vascular meniscal cells (mixed and progenitor) (*p* < 0.0001). Both MChs and PChs had the highest production level of GAGs across all of the cell fractions analysed. Progenitor cells formed firmer and more densely populated fibrochondrogenic pellets in terms of their matrix and cell distribution density compared with mixed population cells across all three tissue types (Figure 8). The higher magnification images (×40) of each cell pellet in Figure 8 were uploaded in supplement material to show more clearly the cell morphology and surrounding matrix. When the immunostaining was assessed semi-quantitively, there was more staining for collagen type I observed in pellets formed by all the progenitor populations compared with the mixed populations, but the difference was not statistically significant (Figure 9A). In contrast, a trend for weaker collagen type II staining was detected in all progenitor pellets compared with mixed population pellets (Figure 9B), while only MChs pellets were found to demonstrate significantly stronger staining for collagen type II compared to PChs pellets (*p* = 0.0383).

## Discussion

Cell-based therapies for meniscus tissue engineering are likely to represent key future meniscus regeneration strategies (Niu et al., 2016). Recent studies have supported the hypothesis that meniscus progenitor cells are the most effective cell type for meniscus regeneration, thought to be due to their tissue specificity and histocompatibility (Muhammad et al., 2014). However, the characteristics of human meniscus progenitor cells have not previously been comprehensively investigated.

Morphologically, the primary progenitors, PAvas and PChs, displayed characteristic cobblestone shaped morphologies, whereas PVas had an elongated fibroblast-like morphology. In the mixed populations, cells presented with more extensive cytoplasmic processes compared to their progenitors. Chondrocytes that display cytoplasmic process could be considered to have undergone a hypertrophic change, which is akin to changes observed in late-stage OA cartilage (Hall, 2019). Samples used in this study were derived from late-stage OA TKR samples, which might explain this distinct morphological feature noted in the mixed populations. However, the progenitor cells isolated from these OA tissues retained a typical proliferative fibroblastic morphology throughout the culture period assessed.

Clonogenicity is a key feature of all types of stem cells derived from various sources including neural (Pitman et al., 2004), hematopoietic (Skobin et al., 2000), embryonic stem cells (Li et al., 2010) and epidermal stem cells (Chan et al., 2004). In our study, progenitor cells from vascular meniscus regions were shown to proliferate at a significantly higher rate compared with the mixed population cells at passages 0–2. Cell-IQ^®^ live cell imaging analyses of colony forming capacity revealed that PAvas, PVas, PChs isolated using fibronectin substrates showed efficient colony forming potential, while the proliferation rates for PVas fractions were significantly faster compared to PAvas and PChs fractions. Seol et al. (Seol et al., 2017) created scratch and punch defects in an explant bovine meniscus, which showed the number of migrated progenitor cells in vascular regions was 8.4 times higher than in avascular regions. This study and our results indicate that PAvas, PVas and PChs possess a capacity for self-regeneration and display stem-like properties. Our results also suggest that vascular meniscal progenitors proliferate faster than avascular meniscal progenitors and chondroprogenitors, which might relate to the higher innate healing potential observed in the vascular meniscus region (Osawa et al., 2013). However, based on the Cell-IQ^®^ live imaging analysis of colony proliferation rates in PAvas, PVas and PChs, not every colony in the fibronectin coated wells showed a superior proliferation capacity. Indeed, 30.3% of colonies were not able to grow beyond 32 cells after 5 days in culture. The cell numbers in these non-progenitor colonies levelled off or even decreased during the 48 h observed. Possible reasons for the presence of non-progenitor colonies might be that some non-progenitor cells which did not attached to fibronectin remained in the wells when media was replenished after 20 min. Additional rounds of PBS washing might help to reduce the number of residual non-progenitor cells, but it also poses a risk of washing away any loosely attached progenitors. To improve the purity of meniscal progenitor cell isolations in the future, cloning rings could be used to isolate monoclonal progenitor cells (Williams et al., 2010), although this technique is more cumbersome and does not lend itself well to good manufacturing practices for translational cell therapy manufacture.

Gamer et al. (Gamer et al., 2017) reported that progenitor cells migrate from murine meniscus explants and express stem cell markers such as CD44, CD90 and CD73. Shen et al. characterised meniscus stem/progenitor cells by seeding the whole meniscus population at a very low density (300 cells/well) and carried out flow cytometry analysis which showed that these cells highly expressed CD44, CD90, CD105, CD166 (Shen et al., 2014). Our flow cytometry data showed that both whole mixed population and progenitor cells from avascular and vascular regions had high expression levels of CD44, CD73, CD90, CD105 and CD166. Thus, CD44, CD73, CD90, CD105 might not be specific to meniscus derived progenitor populations. However, CD166 (Activated-Leukocyte Cell Adhesion Molecule) showed 100% positivity on progenitor populations, while only 90% positivity for MVas and 70% positivity for MChs. Brinkhof et al. (Brinkhof et al., 2020) also identified CD166 as the candidate marker for the identification of bone marrow MSCs (BM-MSCs) in contrast to fibroblasts. Another study showed that vascular meniscal cells express higher CD166 levels compared with avascular meniscal cells in the healthy human meniscus (Grogan et al., 2017). They also found that CD166 positive cells were predominately located in the perivascular, superficial layer and in the adjacent synovial and adipose tissue. Together, this data indicates that CD166 might be an ideal marker for discriminating between progenitors and mixed populations in meniscus and cartilage. Our data also showed that progenitor cells had a higher expression level of integrins including CD49b, CD49c compared to the mixed populations from avascular and vascular regions, as well as chondrocytes from donor-matched cartilage. Integrins are cell membrane receptors associated with cell adhesion and recognition that are essential to cell-cell and cell-matrix interactions (Buckley and Filer, 2017). CD29 (integrin β1) is the most abundant integrin expressed by chondroprogenitor cells (Koelling et al., 2009). CD105+ BM-MSCs subpopulations with high expression levels of CD29 have been shown to have a superior chondrogenic capacity compared with cells that expression a lower percentage of CD29 (Cicione et al., 2010). A previous study also demonstrated that BM-MSCs have high expression levels of CD49c (Jayasuriya et al., 2018). Together, these findings support our results which indicate that integrin marker expression levels could be used to identify progenitor cells in meniscus tissues. In terms of our flow cytometry data the differences noted between avascular and vascular meniscal cells observed were consistent with the findings in our previous study, in that avascular meniscal cells had a higher expression level of the integrin markers CD49b and CD49c compared to vascular meniscal cells from n = 10 donor-matched meniscus tissues (Wang et al., 2020). Other studies have suggested several other potential meniscus progenitor markers. Sun et al. used single -cell RNA sequencing to identify the fibrochondrocyte progenitors (FCP) within healthy human meniscus, which highly express CD146. They further proved CD146+ meniscal cells multilineage differentiation and formed colonies capacity (Sun et al., 2019). Ding et al. derived meniscus MSCs and bone marrow MSCs from rabbits, which both expressed stem cell markers (SSEA-4, Nanog, nucleostemin, strol-1, CD44 and CD90), while meniscus MSCs express less CD34 than bone marrow MSCs(Ding and Huang, 2015). Another study showed similarly to ours that freshly digested meniscal cells from white-white, white-red, red-red zones in healthy human meniscus showed the presence of CD44^+^, CD105+, CD29^+^, CD90^+^ with high prevalence in the WW zone. Interestingly, they also identified CD31^+^ (vasculature marker) cells in all three zones using 3D imaging. Therefore, these extra markers could be potential targets to locate progenitor cells within meniscus tissues.

Previous studies to have investigated meniscal progenitor cells have not reached a consensus on a standard progenitor cell isolation protocol. Table 3 summarizes the meniscus progenitor cell isolation protocols used across the literature. The table shows that the procedures used vary widely from FACS (fluorescence-activated cell sorting) sorting, selective adhesion, low seeding density and tissue explant isolation. All of the proposed methodologies have successfully produced colonies. However, which of these protocols produced progenitor cells with a higher colony forming efficiency or purity is unclear. The basic principles of the progenitor cells isolation protocol used in this study are based on the selection of cells that highly express β1 integrins and demonstrate rapid adhesion to extracellular matrix proteins (Jones and Watt, 1993), which was originally used for chondroprogenitors isolation (Williams et al., 2010). KorSimilar et al. (Korpershoek et al., 2021) and Pattappa et al. (Pattappa et al., 2022) also used the fibronectin selective protocol to isolate meniscus progenitor cells. However, we included a direct comparison of the protocol for the efficiency of donor-matched chondrocyte, avascular and vascular meniscus progenitor isolation, and present these results for the first time (to our knowledge).

**TABLE 3:** Published meniscal progenitor cells isolation protocols.

References	Tissue source	Progenitor cells isolation protocol
Ding and Huang, (2015)	Rabbit Meniscus	Suspended digested meniscal cells in growth media and cultured for 10–15 days to form colonies
Huang et al. (2016)	Rabbit Meniscus	Suspended digested meniscal cells in stem cell growth media and cultured for 8–10 days to form progenitor colonies
Seol et al. (2017)	Bovine Meniscus	Progenitor cells that had migrated into injured sites *in vitro* were isolated followed by trypsin and collagenase digestion
Shen et al. (2014)	Bovine Meniscus	Meniscal cells were seeded at a very low density to form colonies (300 cells in one 6 cm dish)
Gamer et al. (2017)	Murine Meniscus	Menisci explant cultures in 50 μL of media for 2 h for tissue adhesion followed by culture in 1.5 ml of media for 3 days with an additional 1.5 ml of media for a further 5–7 days. Meniscal progenitor cells migrated from explant tissues
Sun et al. (2019)	Healthy Human Meniscus	Single cell RNA-seq identified the fibrochondrocyte progenitor set highly express CD146, CD146+ meniscal cells selected by flow cyctometry
Chahla et al. (2021)	Healthy Human Meniscus	Fresh digested meniscus stromal progenitor cells in were characterized by flow cytometry for MSC surface markers (CD105, CD90, CD44, CD29)
Osawa et al. (2013)	Meniscus from aborted human fetuses and TKRs	FACS was used to isolate CD34 and CD146 positive meniscal cells after meniscus tissue digestion
Korpershoek et al. (2021)	Human Meniscus from TKRs	A total of 500 cells/cm^2^ were plated on the fibronectin-coated flasks and non-adherent cells were removed after 20 min
Pattappa et al. (2022)	Human Meniscus from TKRs	Meniscal cells were seeded at 2 × 10^3^ cells/ml (200 cells/cm^2)^ onto fibronectin-coated plates and incubated for 20 min in either a standard cell culture incubator at 20% oxygen and 5% CO_2_ or a low oxygen incubator

TKR: total knee replacement; FACS: fluorescence-activated cell sorting.

In the chondrogenic analyses undertaken in this study, the immunochemistry histological staining of chondrogenic pellets demonstrated that meniscal progenitor cells were more chondrogenic compared with the mixed population cells. In addition, we found that progenitor cells generally produced higher amounts of GAGs compared with mixed population cells in terms of GAG/DNA analyses. These findings suggest that the progenitor population in the meniscus is a suitable cell source for use in rebuilding the proteoglycan-rich avascular zone of damaged menisci, which represents a key challenge for meniscus repair in the clinic (Shimomura et al., 2018). Interestingly, we found that the collagen type II staining intensity of PChs was significantly lower than MChs. The downregulation of collagen type II is seen as a sign of chondrocyte dedifferentiation (Benya, 1982). Typically, *in vitro* cultured human articular chondrocytes become dedifferentiated and lose their ability to produce hyaline cartilage tissue as their passage number increases (Lee et al., 2017). The dedifferentiation phenomenon of PChs may be caused by the inclusion of FGF2 growth factor in their culture media which is aimed at stimulating the proliferation of progenitor cells. Lee et al. (Lee et al., 2017) cultured costal chondrocytes *in vitro* with or without FGF2 supplementation and demonstrated that the addition of FGF2 accelerated cell expansion and dedifferentiation. However, chondrocytes cultured with FGF2 supplementation showed better fibrochondrogenic differentiation potential both *in vitro* and *in vivo* compared to chondrocytes cultured without FGF2. Therefore, the addition of FGF2 to culture media may have induced a rapid but reversible dedifferentiation during the *in vitro* expansion phase. Another possible explanation for the low collagen type II staining observed could be that the samples involved in this study were all derived from OA environment, which was previously reported to induce a gradual loss of collagen type II and a decrease in mRNA expression of COL2A1 in degenerate human cartilage tissues (Zhong et al., 2016).

Two major limitations were noted in this study. The first limitation being that the meniscus samples used were all derived from OA donors, which may not truly reflect the healthy meniscal progenitor cells from normal tissue. The inflammatory environment in some OA joints could activate cytokines e.g. interleukin 1, which may affect the biological behavior of meniscus progenitor cells. Another limitation was the sample size of this study, which was quite limited (n = 5) and might lead to the inaccurate results.

In conclusion, our study demonstrates that the human meniscus contains meniscal progenitor populations in both the avascular and vascular regions based on clonogenicity and chondrogenic differentiation capacity. Our results also suggested that meniscal progenitors derived from the vascular region of the meniscus exhibit enhanced reparative characteristics which likely associate with the better meniscal healing potential previously observed in the vascular region. The findings of this study build on the body of evidence which suggests that meniscal progenitors represent an attractive cell therapy strategy for the enhancement of meniscal repair and regeneration.

## Data Availability

The original contributions presented in the study are included in the article/Supplementary Material, further inquiries can be directed to the corresponding authors.
